# Legal Phase of X-Rays

**Published:** 1915-10

**Authors:** 


**Legal Phase of X-Rays.** N. C. Morse of Eldorado, Iowa, Railway Surg. Jour., May, discusses the danger to surgeons from suits based on X-ray pictures which often show a considerable deformity in spite of good functional recovery and especially because such pictures do not make allowance for difficulties inherent to the case.

